# Laparoscopic versus open repair of perforated peptic ulcers: analysis of outcomes and identification of predictive factors of conversion

**DOI:** 10.1007/s13304-022-01391-6

**Published:** 2022-10-03

**Authors:** Dario Tartaglia, Silvia Strambi, Federico Coccolini, Alessio Mazzoni, Mario Miccoli, Camilla Cremonini, Enrico Cicuttin, Massimo Chiarugi

**Affiliations:** 1 Emergency Surgery Unit, New Santa Chiara Hospital, University of Pisa, Via Paradisa, 2, 56021 Pisa, Italy; 2 Department of Clinical and Experimental Medicine, University of Pisa, Pisa, Italy

**Keywords:** Conversion to open surgery, Duodenum, Laparoscopy, Perforated peptic ulcer, Stomach

## Abstract

**Background:**

The surgical treatment for perforated peptic ulcers (PPUs) can be safely performed laparoscopically. This study aimed to compare the outcomes of patients who received different surgical approaches for PPU and to identify the predictive factors for conversion to open surgery.

**Methods:**

This retrospective study analyzed patients treated for PPUs from 2002 to 2020. Three groups were identified: a complete laparoscopic surgery group (LG), a conversion to open group (CG), and a primary open group (OG). After univariate comparisons, a multivariate analysis was conducted to identify the predictive factors for conversion.

**Results:**

Of the 175 patients that underwent surgery for PPU, 104 (59.4%) received a laparoscopic-first approach, and 27 (25.9%) required a conversion to open surgery. Patients treated directly with an open approach were older (*p* < 0.0001), had more comorbidities (*p* < 0.0001), and more frequently had a previous laparotomy (*p* = 0.0001). In the OG group, in-hospital mortality and ICU need were significantly higher, while the postoperative stay was longer. Previous abdominal surgery (OR 0.086, 95% CI 0.012–0.626; *p* = 0.015), ulcer size (OR 0.045, 95% CI 0.010–0.210; *p* < 0.0001), and a posterior ulcer location (OR 0.015, 95% CI 0.001–0.400; *p* = 0.012) were predictive factors for conversion to an open approach.

**Conclusion:**

This study confirms the benefits of the laparoscopic approach for the treatment of PPUs. Previous laparotomies, a greater ulcer size, and a posterior location of the ulcer are risk factors for conversion to open surgery during laparoscopic repair.

## Introduction

Perforated peptic ulcers (PPUs) are the second most frequent cause of abdominal perforations and the leading indication for emergency gastric surgery. Among the peptic ulcer complications, perforation is associated with short-term mortality and morbidity in up to 30 and 50% of patients, respectively, due to secondary peritonitis and sepsis [1]. As surgical delays significantly increase mortality, immediate surgery is the key point of therapy [2, 3].

The best surgical approach for the definitive treatment of this pathology has been discussed for decades. However, it has not yet been possible to develop strong guidelines [4, 5].

In the 1990s, laparoscopic repair of PPUs was first described [6]. Laparoscopy allows for minimally invasive detection and closure of the lesion with adequate peritoneal lavage, without the drawbacks of an upper laparotomy. Less postoperative pain and analgesic consumption, shorter recovery durations, and decreased wound infections are just some of the advantages of laparoscopic repair [7, 8]. However, a non-negligible number of patients require conversion from a laparoscopic approach to open surgery. Others instead require a primary open surgery, as they may not be fit for laparoscopy, or the surgeons may not feel comfortable with this minimally invasive technique.

This study primarily aimed to identify the predictive factors for conversion to open surgery and secondarily to compare the clinical characteristics, intraoperative findings, and outcomes of patients undergoing different surgical approaches for PPUs.

## Methods

### Study design

The charts of patients undergoing laparoscopic surgery for PPUs at a single tertiary center from 2002 to 2020 were retrospectively analyzed. In these cases, a suspicion of PPU was raised after clinical evaluation and all patients underwent a CT scan with contrast to confirm the presence of a PPU. All operations were performed or supervised by experienced surgeons. The Institutional Review Board of New Santa Chiara Hospital approved the study. This study is compliant with the ethical standards of the 1964 Helsinki declaration and its later amendments or comparable ethical standards. Informed consent for this study was waived because of its retrospective nature.

### Surgical procedures

In all patients undergoing laparoscopic repair, a “French” position with a reverse Trendelenburg tilt was adopted. Pneumoperitoneum was established using a Veress needle or according to Hasson’s open technique. Trocars were placed at the umbilicus (for the videoscope, size 12 mm) and on the left and the right midclavicular line above the umbilicus level (for the instruments, size 5 mm). If necessary, a fourth trocar was placed in the subxiphoid space for lavage or liver retraction. A 5 or 10 mm 30° videoscope was used during the surgery.

After a full abdominal exploration, the supramesocolic region was meticulously assessed for perforation. A cautious blunt adhesiolysis was adopted in the case of significant inflammatory adhesions. Instrumental compression of the stomach's antrum and the first part of the duodenum facilitated the identification of the perforation by inducing the escape of fluid and bubbles. Closure of the PPU was achieved by absorbable interrupted stitches using an intracorporeal knotting technique, and the omentum was secured above the repaired perforation. An air or methylene blue test was performed to rule out leakage from the suture. Irrigation with a saline solution was done throughout the peritoneal cavity until a complete cleaning was achieved and, at the end of the procedure, drains were put in place. For laparotomies, the closure of the ulcer was carried out with a double-layer interrupted stitch with omentopexy. According to the preferences of the attending surgeon, gastric resections were followed by a Roux-en-Y reconstruction or Billroth II.

### Data collection and statistics

**Fig. 1:**
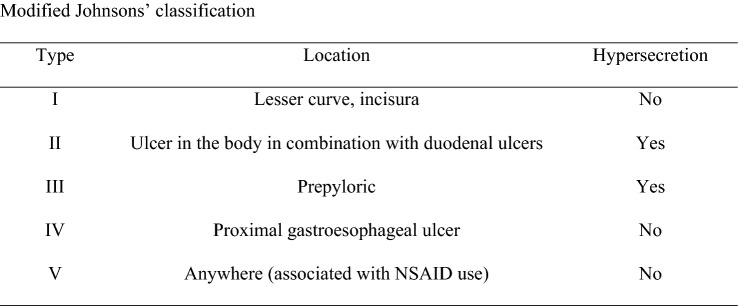
Modified Johnsons’ classification

For the analysis, three groups of patients who underwent operations for PPUs were identified: (1) a total laparoscopy group (LG), (2) a converted to open group (CG), and (3) a primary open group (OG). The following parameters were analyzed: gender, age, body mass index (BMI), comorbidities, pre-existing proton pump inhibitor (PPI) treatment, smoking status, previous abdominal surgery, American Society of Anesthesiology (ASA) score, Mannheim Peritonitis Index (MPI), Charlson Comorbidity Index, time between clinical presentation and treatment, Boey’s score, ulcer location (stomach vs. duodenum), modified Johnson’s classification (Fig. 1) [9], ulcer side (anterior vs. posterior), ulcer size, surgical procedures performed, conversion to open surgery, operative time, in-hospital morbidity, re-intervention rate, in-hospital mortality, ICU need, length of ICU stay, and length of postoperative stay.

The primary endpoint of the study was the conversion rate to open surgery. Secondary endpoints were in-hospital morbidity, reoperation, in-hospital mortality, ICU admission rate, ICU LOS, and postop LOS.

Descriptive statistics are presented as mean ± standard deviation and median with interquartile range. Shapiro–Wilk test was performed to verify the normality of the quantitative distributions. Data were not normally distributed. Kruskal–Wallis test, Mann–Whitney *U* test, Chi-squared test and Fisher’s exact test were used to compare different groups.

Multivariable binary logistic regression was carried out to analyze the relationship between the predictor factors and the outcome. A *p* value ≤ 0.06 was the threshold to select the independent variables for the multivariable model. The results of the model are presented as an odds ratio (OR) with the associated 95% confidence interval. A *p* value < 0.05 was considered statistically significant.

Statistical analysis was performed using XLSTAT software (Addinsoft, Chicago, IL, USA) and R 4.0.3.

## Results

Between 2002 and 2020, 175 patients underwent surgery for PPUs. Overall, 96 patients (54.8%) were male, the mean age was 64.5 (± 19.7) years, and the mean BMI was 24.2 (± 4.3) kg/m^2^ (Table 1). Comorbidities were found in 120 cases (68.6%) and 23 patients (13.1%) were taking PPIs prior to the operation. In 46 cases (26.2%), the patients had had a previous abdominal surgery. Prior to surgery, the median ASA score was 3 (IQR 1–4), the mean MPI 20.6 (± 6.2), the mean Charlson comorbidity index 3.1 (± 2.4), the mean time clinical presentation/treatment 31.7 (± 23.8) hours and mean Boey’s score was 1.2 (± 0.8).

**Table 1:** Preoperative characteristics of patients with perforated peptic ulcers treated with various surgical approaches

	Overall *n* = 175	Laparoscopy group *n* = 77	Converted to open group *n* = 27	Open group *n* = 71	*p* value LG vs. CG	*p* value CG vs. OG	*p* value LG vs. OG
Male gender, *n* (%)	96 (54.8)	48 (62.3)	16 (59.2)	32 (45)	0.95	0.30	0.05
Age, years, mean (± SD)	64.5 (± 19.7)	55.9 (± 19.4)	65.5 (± 14.1)	73.5 (± 17.49)	0.05	**0.01**	** < 0.0001**
BMI, kg/m^2^, mean (± SD)	24.2 (± 4.3)	23.9 (± 3.2)	25.3 (± 4.6)	24.7 (± 4.9)	0.12	0.50	0.24
Co-morbidities, *n* (%)	120 (68.6)	40 (51.9)	20 (74)	60 (84.5)	0.26	0.36	** < 0.0001**
Pre-existing PPI treatment, *n* (%)	23 (13.1)	8 (10.3)	2 (7.4)	13 (18.3)	0.20	0.22	0.25
Smoking status, *n* (%)	74 (42.2)	32 (41.5)	15 (55.5)	27 (38)	0.62	0.18	0.78
Previous abdominal surgery, *n* (%)	46 (26.2)	9 (11.6)	8 (29.6)	29 (40.8)	**0.02**	0.43	**0.0001**
Charlson comorbidity index, mean (± SD)	3.1 (2.4)	1.9 (2.1)	3 (1.7)	4.8 (2.1)	**0.03**	**0.02**	** < 0.0001**
Time presentation/treatment (hs) mean (± SD)	31.7 (23.8)	30.9 (24.4)	28.4 (21.9)	33.8 (23.9)	0.66	0.26	0.34
Boey score mean (± SD)	1.2 (0.8)	0.8 (0.6)	1.4 (0.7)	1.6 (0.8)	**0.003**	0.25	** < 0.0001**
ASA score, median (IQR)	3 (1–4)	2 (2–3)	2 (1.9–2.3)	3 (3.1–3.4)	0.29	0.23	0.06
Mannheim peritonitis index, mean (± SD)	20.6 (± 6.2)	19.1 (5.2)	21.8 (6.4)	21.6 (6.9)	**0.04**	0.89	0.05

Bolding indicates statistically significant differences (*p* < 0.05)

*ASA* American Society of Anesthesiology score *BMI* Body Mass Index, *PPI* Proton Pump Inhibitor, *SD* Standard Deviation, *IQR* Interquartile Range, *y* years

**Fig. 2:**
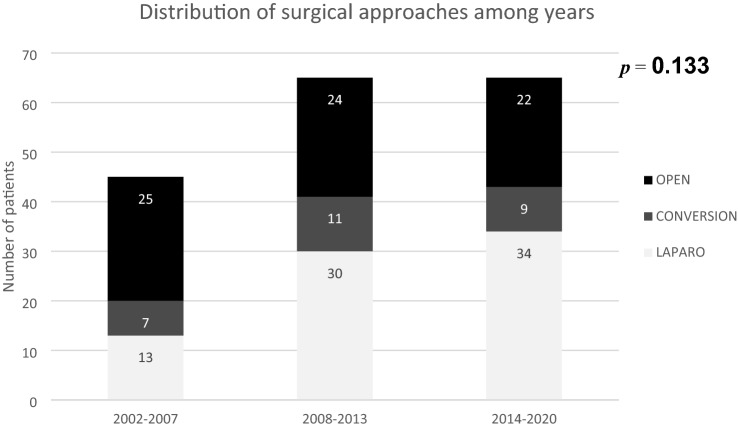
Distribution of surgical approaches among years

A complete laparoscopic treatment was performed in 77 patients (44%), conversion to an open approach was necessary in 27 cases (15.4%), and 71 patients (40.6%) were treated with a primary laparotomy. Over the years, the distribution of the surgical approach did not significantly differ (Fig. 2). In 61.1% of cases, the ulcer was located in the stomach. According to the modified Johnson’s classification, which was applied to 109 patients, 35 (32%) were classified as type I, 2 (1.8%) as type II, 53 (48.6%) as type III, 1 (0.9%) as type IV, and 18 (16.5%) as type V. The mean ulcer size was 12.3 mm (± 9.2). In 88% of cases, the ulcer was located anteriorly. The reasons for conversion to an open approach were inadequate ulcer identification in nine patients (33%), the large size of the perforation in six patients (22%), severe inflammatory involvement of surrounding tissues in five patients (19%), adhesions in four patients (15%), and a suspected tumor in three patients (11%). Overall, the most commonly performed procedure was suture with omentopexy (88%), followed by gastric resection with Roux-en-Y reconstruction (7.4%), Billroth II (2.8%), and omentopexy only (1.8%). Gastric resections with Roux-en-Y reconstruction or Billroth II were performed only in CG and OG patients. The mean operative time was 119.4 (± 68.8) min. Fifty-six patients (32%) required intensive care support in the immediate postoperative course and the mean ICU stay was 6.6 (± 10) days. In-hospital morbidity was 47.7% (73/153) and 10 patients (5.7%) required a re-intervention. Death occurred in 22 patients (12.5%). Overall, the mean postoperative stay was 10.7 (± 8) days.

As shown in Table 1, males were marginally more frequent in the LG (62.3%) than the OG group (45%; *p* = 0.05). Mean age appeared increasingly older from LG to OG (55.9 ± 19.4, 65.5 ± 14.1, and 73.5 ± 17.49 years, respectively; LG vs. CG: *p* = 0.05; CG vs. OG: *p* = 0.01; LG vs. OG: *p* < 0.0001). No significant differences were found between the three groups in terms of BMI, pre-existing PPI treatment, smoking status or ASA score (Table 1). Comorbidities were significantly less frequent in the LG group as compared to the OG group (51.9 vs. 84.5%; *p* < 0.0001). The rate of previous abdominal surgeries was significantly higher in the CG (29.6%) and OG (40.8%) groups as compared to the LG group (11.6%; LG vs. CG: *p* = 0.02; LG vs. OG: *p* = 0.0001). The MPI was also significantly higher in the CG (21.8 ± 6.4) and OG (21.6 ± 6.9) groups as compared to the LG group (19.1 ± 5.2; LG vs. CG: *p* = 0.04; LG vs. OG: *p* = 0.05). Mean Charlson Comorbidity Index was significantly higher in CG (3 ± 1.7) and OG (4.8 ± 2.1) than LG (1.9 ± 2.1). No significant difference was found about time spam elapsed between presentation and treatment. Mean Boey’s score was significantly higher in LG vs CG (*p* = 0.003) and LG vs OG (*p* < 0.0001).

Differences in ulcer location between the stomach and duodenum were more evident when comparing the LG and OG groups (74% vs. 26% and 47.8% vs. 52%, respectively; *p* = 0.002; Table 2). According to the modified Johnson’s classification for stomach ulcer distribution, there were significant differences between the LG vs. the CG and OG groups (*p* = 0.008 and *p* < 0.001, respectively). However, there were no significant differences between the CG and OG groups on this measure (*p* = 0.49; Table 2). Conversely, a posterior side location for the ulcer was more frequent in the CG (37%) and OG (15.4%) groups than in the LG group (LG vs. CG: *p* < 0.0001; CG vs. OG: *p* = 0.04; LG vs. OG: *p* = 0.0002). The same pattern of results was observed for mean ulcer size, which was 7.1 ± 3.9 mm in LG, 19.1 ± 9.8 mm in CG, and 15.2 ± 10 mm in OG (LG vs. CG: *p* < 0.0001; CG vs. OG: *p* = 0.03; LG vs. OG: *p* < 0.0001). Patients converted to an open surgery required a longer operative time (180.6 ± 84.9 min) compared to the LG (89.6 ± 21.3 min) and OG (128.7 ± 73.6 min) groups (LG vs. CG: *p* < 0.0001; CG vs. OG: *p* = 0.001; LG vs. OG: *p* = 0.001; Table 2).

**Table 2:** Intraoperative findings in patients with perforated peptic ulcers treated with various surgical approaches

	Overall *n* = 175	Laparoscopy group *n* = 77	Converted to open group *n* = 27	Open group *n* = 71	*p* value LG vs. CG	*p* value CG vs. OG	*p* value LG vs. OG
Ulcer location, *n* (%)							
Stomach	107 (61.1)	57 (74)	16 (59.2)	34 (47.8)	0.23	0.43	**0.002**
Duodenum	68 (38.9)	20 (26)	11 (40.7)	37 (52)			
Modified Johnson’s classification, *n* (%)					**0.008**	0.49	** < 0.0001**
I	35/109 (32)	8/57 (14)	7/16 (43.7)	20/36 (55.5)			
II	2/109 (1.8)	0	0	2/36 (5.5)			
III	53/109 (48.6)	38/57 (66.6)	5/16 (31.2)	10/36 (27.7)			
IV	1/109 (0.9)	0	1/16 (6.2)	0			
V	18/109 (16.5)	11/57 (19.3)	3/16 (18.7)	4/36 (11)			
Ulcer side, *n* (%)							
Anterior	154 (88)	77 (100)	17 (63)	60 (84.5)	** < 0.0001**	**0.04**	**0.0002**
Posterior	21 (12)	0	10 (37)	11 (15.4)			
Ulcer size, mm, mean (± SD)	12.3 (± 9.2)	7.1 (3.9)	19.1 (9.8)	15.2 (10)	** < 0.0001**	**0.03**	** < 0.0001**
Surgical procedures, n (%)							
Suture with omentopexy	154 (88)	74 (96)	20 (74)	60 (84.5)	**0.001**	0.26	**0.01**
Gastric resection with Roux-en-Y reconstruction	13 (7.4)	0	5 (18.5)	8 (11.2)	**0.001**	0.33	**0.002**
Billroth II	5 (2.8)	0	2 (7.4)	3 (4.2)	0.06	0.30	0.22
Omentopexy only	3 (1.8)	3 (4)	0	0	0.56	1	0.24
Operative time, min, mean (± SD)	119.4 (± 68.8)	89.6 (21.3)	180.6 (84.9)	128.7 (73.6)	** < 0.0001**	**0.001**	**0.001**

Bolding indicates statistically significant differences (*p* < 0.05)

*SD* Standard Deviation

The in-hospital morbidity was significantly higher in the CG (63.6%) and OG (70.6%) groups as compared to the LG group (24.6%; LG vs. CG: *p* = 0.005; CG vs. OG: *p* = 0.76; LG vs. OG: *p* < 0.0001; Table 3). However, there were no significant differences in the re-intervention rate between the three groups (Table 3). The reasons for re-interventions were leakage in 6 cases (LG: 2; CG: 1; OG: 3), purulent collections in three cases (LG: 2; OG: 1), and wound dehiscence (OG: 1). There were four deaths in the LG group (5.2%), 5 (5.2%) in the CG group, and 13 (18.3%) in the OG group. However, the difference in the number of deaths was significant only between LG and OG (*p* = 0.02; Table 3). The need for ICU stays was increasingly and significantly higher from the LG to the OG group (2.6, 29.6 and 64%, respectively; LG vs. CG: *p* < 0.0001; CG vs. OG: *p* = 0.04; LG vs. OG *p* < 0.0001; Table 3). Accordingly, a similar pattern of results was observed for the mean postoperative stay (LG vs. CG: *p* = 0.01; CG vs. OG: *p* = 0.13; LG vs. OG: *p* < 0.0001; Table 3).

**Table 3:** Postoperative comparisons of patients with perforated peptic ulcers treated with various surgical approaches

	Overall *n* = 175	Laparoscopy group *n* = 77	Converted to open group *n* = 27	Open group *n* = 71	*p* value LG vs. CG	*p* value CG vs. OG	*p* value LG vs. OG
In-hospital morbidity, *n* (%)	73/153 (47.7)	18/73 (24.6)	14/22 (63.6)	41/58 (70.6)	**0.005**	0.76	** < 0.0001**
Re-intervention, *n* (%)	10 (5.7)	4 (5.2)	1 (3.7)	5 (7)	0.57	0.99	0.73
In-hospital mortality, *n* (%)	22 (12.5)	4 (5.2)	5 (5.2)	13 (18.3)	0.23	0.79	**0.02**
ICU need, *n* (%)	56 (32)	2 (2.6)	8 (29.6)	46 (64.7)	** < 0.0001**	**0.04**	** < 0.0001**
ICU stay, days mean (± SD)	6.6 (10)	2.5 (0)	7.9 (6.2)	6.6 (10.6)	0.50	0.74	0.58
Postoperative stay, days, mean (± SD)	10.7 (± 8)	7.3 (2.6)	11.6 (6.6)	14.1 (10.5)	**0.01**	0.13	** < 0.0001**

Bolding indicates statistically significant differences (*p* < 0.05)

*ICU* Intensive Care Unit, *SD* Standard Deviation

For the multivariate analysis, a previous abdominal surgery (OR 0.086, 95% CI 0.012–0.626; *p* = 0.015), ulcer size (OR 0.045, 95% CI 0.010–0.210; *p* < 0.0001), and a posterior ulcer location (OR 0.015, 95% CI 0.001–0.400; *p* = 0.012) were identified as predictive factors for a conversion to open surgery (Table 4). The *R*^2^ (Nagelkerke) was 0. 946, indicating an appropriate level of goodness of fit.

**Table 4:** Multivariate analysis for the evaluation of predictive factors of conversion from a laparoscopic to an open approach

Variables	*p* value	Odds ratio	Odds ratio lower limit (95% CI)	Odds ratio upper limit (95% CI)
Age	0.448	1.033	0.950	1.124
Charlson comorbidity index	0.598	0.827	0.408	1.677
Boey’s score	0.069	0.266	0.064	1.110
Mannheim peritonitis index	0.635	1.038	0.891	1.209
Previous abdominal surgery (yes vs. no)	**0.015**	**0.086**	**0.012**	**0.626**
Ulcer size (cm)	** < 0.0001**	**0.045**	**0.010**	**0.210**
Ulcer location (posterior vs. anterior)	**0.012**	**0.015**	**0.001**	**0.400**
Modified Johnson’s Classification				
I				
II	0.945	0.919	0.084	10.017
III	0.160	6.342	0.483	83.244
V	0.308	4.468	0.251	79.638

Bolding indicates statistically significant factors (*p* < 0.05)

*R*^2^ (Nagelkerke) = 0.946

## Discussion

We report here that a larger ulcer size, a posterior location, and the presence of previous laparotomies are strong predictive factors for conversion to open surgery in patients with PPUs treated laparoscopically. Furthermore, we confirm previous findings showing that laparoscopy is related to lower morbidity rates, lower mortality, and shorter postoperative hospital stays, and that patients treated with a primary open approach are significantly more impaired.

Despite the long interval time of the study (2001–2020), we found that there was no significant difference in terms of distribution of the surgical approaches over the years (Fig. 2). This finding remarks a sort of continuity of the operative unit in establishing the type of approach that should be dedicated to patients with PPUs. In fact, neither organizational nor logistic factors nor modifications of clinical protocols have been significantly changed all over years. That surgical constancy gives more significance to the results reported in the present study.

Given the low incidence of PPUs in the general population, determining the most accurate surgical approach for this condition has been very challenging [10]. However, it has been demonstrated that laparoscopy is a safe, feasible, and effective approach for PPUs, when not contraindicated [11–13]. The principal surgical technique for repairing PPUs has been another topic of debate and several methods have been described in the literature [4, 14], including primary closure of the perforation through interrupted sutures with or without an overlay and an omental pedicle, and sutureless repair by occluding the perforation with a pedicled omentoplasty (Cellan-Jones repair) [15] or by placing a free omental patch (Graham patch as originally described) [16]. While recent evidence has suggested that ulcer repair by closing the perforation with absorbable interrupted stitches, omentopexies, and lavage of the abdominal cavity is the more effective surgical strategy for PPUs smaller than 2 cm [17], no strong recommendations have been made [5].

Several studies, including randomized controlled trials and meta-analyses, have outlined the advantages of laparoscopic repair over an open approach for treating PPUs [18]. A recent meta-analysis included six randomized controlled trials examining 319 patients that received a laparoscopic repair and 312 that received an open repair [13]. It was reported that LR patients needed a conversion due to technical difficulties (9.4%), the size of perforation (57.1%), extensive peritoneal adhesions (21.4%), hemodynamical instability (17.9%), or failure to find the site of perforation (7.1%). The overall morbidity (8.9 vs. 17%) and wound infection rate (2.2 vs. 6.3%) were significantly lower in the LR group compared to OR group, while there were no significant differences in terms of post-operative leakage (1.1 vs. 0.3%), intraoperative abscess (1 vs. 1.7%), postoperative sepsis (0.8 vs. 0.5%), postoperative ileus (0.6 vs. 1.4%), incisional hernia (0 vs. 6%), re-operation rate (1.1 vs. 0.5%), or mortality rate (1 vs. 1.4%). Furthermore, patients undergoing LR had a significantly shorter length of stay in hospital. Thus, the authors concluded that the laparoscopic approach should be considered the treatment of choice for PPUs [13]. However, no specific attention was paid in this study to patients who required conversion to an open approach, as they were analyzed within the LR group, creating a potential bias in the results. In accordance with Quah et al., we also found that morbidity was significantly lower in the LG group compared to the CG and OG groups, the postoperative stay was significantly longer in the OG group, while there were no significant differences in terms of the re-intervention rate. Mortality and ICU need were significantly higher in the OG group, reflecting the preoperative impairments affecting patients treated primarily with an open approach.

Despite the evidence supporting the use of laparoscopic repair, deciding when to convert to an open surgery is still a matter of debate [19] in the current study, laparoscopic repair of PPU was adopted in 59.4% of all patients, with a conversion rate of 25.9%, which is in line with the rates reported in the literature (10.4–52.7%) [20–26]. The main reasons for conversion were a posterior location that did not allow for proper inspection of the ulcer (33%), a large perforation size (22%), severe flogystic involvement of the surrounding tissues (19%), adhesions (15%), and suspected tumors (11%). The listed reasons of conversion are in line with those reported in the literature [13]. As previously stated by Muller et al., we confirm that conversion to an open approach can only be assessed intraoperatively [23]. It should be noted that experience in laparoscopy may be a crucial factor that contributes to the conversion rate. In the present case series from our high-volume center, the high percentage of PPU patients treated with laparoscopy (55.9%) and the relatively low percentage of conversion (25.9%) could be due to a high degree of laparoscopic surgical experience at our center. An improved laparoscopic “background” may also contribute to better outcomes for patients. Indeed, the overall morbidity (47.7%) and mortality (12.5%) rates in the current study are lower than that described in the literature (60 and 21.2%, respectively) [13, 26]. However, almost 40% of patients with PPU were performed with a primary open approach in our institution, which represents a relevant quote of cases for the study. The reasons could be identified in the general characteristics of those patients and their related general hemodynamical conditions, which did not allow them to safely undergo laparoscopic surgery. In fact, a significantly higher rate of comorbidities (84.5%), previous abdominal surgery (41%), hemodynamical instability (*n* = 21/71, 30%), and an important grade of patients’ compromise highlighted by a significantly higher ASA score (3 [3.1–3.4]), Mannheim Peritonitis Index (21.6 ± 6.9), Charlson Comorbidity index (4.8 [2.1]), and Boey’s score (1.6 [0.8]) justified the surgeons’ decision to make an open surgery.

Ulcer size is one of the most important factors leading to conversion to an open surgery. While there is no consensus regarding the exact diameter necessary for conversion, a perforation smaller than 10 mm can be easily repaired in laparoscopy. In contrast, larger perforations can be associated with adjacent tissue infiltration and fragility, making the repair more difficult [27]. In their analysis of 77 PPU patients approached laparoscopically, Kim et al. reported that ulcer perforation size was the only risk factor for conversion. A mean perforation size of 14 mm was observed in converted patients, which was significantly greater than that seen in the laparoscopic repair group (3.8 mm) [20]. Similarly, Lunevicius et al. also showed that perforated ulcers were significantly larger in converted patients (6.0 mm) than in a completed laparoscopy group (3.5 mm), and ulcer size was the only significant predictive factor identified for conversion [22].

Muller et al. also reviewed 36 patients undergoing laparoscopy for perforated gastric ulcer and identified a larger mean perforation size in the converted group (3.9 vs. 10.2 mm); however, multivariate analysis in this study did not identify perforation size as a significant predictive factor for conversion [23]. In the present study, we identified a significantly greater mean perforation size (7.1 vs. 19.1 mm) in the CG group and the size of the perforation was a strong predictor of conversion, in line with that reported by Kim et al. [20]. Based on this finding, we agree with Kumar et al., who suggested a conversion to open surgery in the presence of an ulcer > 2 cm to allow for an eventual resection if the patient’s clinical condition allows it [28].

Interestingly, the current study showed that neither the gastric or duodenal location of the ulcer, nor the modified Johnson’s classification, were predictive factors for a conversion to an open approach. This finding is substantially in line with data reported by Zimmermann et al. who examined a cohort of 45 laparoscopically approached PPU patients [26]. However, a posterior location of the perforation was found to be more frequent in the CG group and was a strong predictive factor of conversion. Previous studies did not focus on this intraoperative aspect [20, 22, 25], but it has been reported that the main reason for conversion to an open surgery is a “difficult” localization of the ulcer [29]. Posteriorly located ulcers can be difficult to detect and inevitably require increased mobilization of the stomach and the duodenum for a clearer view. Increased mobilization of these structures is typically achieved by opening the gastrocolic ligament, takedown of the right colonic flexure, and a Kocher maneuver. There is no doubt that all these procedures are quite demanding when using a laparoscopic approach, and even more so in emergency settings.

Another important observation from the current study is that a history of previous abdominal surgery is a predictive factor for the conversion to an open approach. As previous studies have tended to include open upper abdominal surgery as an exclusion factor, it is quite difficult to compare this finding with the literature. Nonetheless, Lunevicious et al. reported that 6.5% of patients (46 total cases) completing laparoscopy had a previous abdominal surgery compared to 8.3% of converted patients (12 cases total), which did not differ significantly [22]. Similarly, Kim et al. did not observe a difference between laparoscopic (*n* = 69) and conversion (*n* = 8) groups in terms of operative history (5.8 vs. 12.5%). Thus, the authors did not consider this parameter a potential risk factor for conversion in their multivariate analysis [20]. In addition, Zimmerman et al. did not observe a significant difference between laparoscopic and conversion groups (4 vs. 5%) [26]. Conversely, in their study of 36 PPU patients treated with a primary laparoscopic approach, Muller et al. identified the presence of adhesions as a “determinant” factor for conversion to open surgery, even though the authors did not provide specific numbers in the multivariate analysis [23]. We strongly suggest performing a conversion when adhesions are not dissectible laparoscopically. Lysing attempts may either increase either the length of the operation time or the risk of visceral injuries, which are inevitably related to higher complication rates in the postoperative course.

There are several limitations to this study. First, the study was conducted retrospectively and at a single institution. Second, some preoperative clinical data, such as the Boey or APACHE II scores, were not included in the analysis as they were missing for most cases. These measures may be potential risk factors for conversion and further investigations are needed to address this possibility. Third, selection of the operative approach was done at the discretion of the on-duty surgeon and may been affected by issues other than the patient’s condition. Despite these limitations, the data obtained from the relatively large cohort of patients enrolled in the current study should prove to be a meaningful contribution to the current literature.

## Conclusion

PPU patients may benefit from a primary laparoscopic surgery when not contraindicated for anesthesiologic reasons. A larger ulcer size, a posterior location, and the presence of adhesions due to previous abdominal surgery may require conversion from a laparoscopic approach to an open surgery to treat the perforation properly. As these factors can only be detected intraoperatively, it is not feasible to preoperatively identify patients at a high risk of conversion.

## Data Availability

Data were retrospectively collected from reports and recorded in a database.

## References

[1] Søreide K, Thorsen K, Harrison EM (2015). Perforated peptic ulcer. Lancet.

[2] Møller MH, Adamsen S, Thomsen RW, Møller AM (2010). Preoperative prognostic factors for mortality in peptic ulcer perforation: a systematic review. Scand J Gastroenterol.

[3] Buck DL, Vester-Andersen M, Møller MH, Danish Clinical Register of Emergency Surgery (2013). Surgical delay is a critical determinant of survival in perforated peptic ulcer. Br J Surg.

[4] Bertleff MJ, Lange JF (2010). Perforated peptic ulcer disease: a review of history and treatment. Dig Surg.

[5] Tarasconi A, Coccolini F, Biffl WL (2020). Perforated and bleeding peptic ulcer: WSES guidelines. World J Emerg Surg.

[6] Mouret P, François Y, Barth X VJ, Lombard-Platet R (1990). Laparoscopic treatment of perforated peptic ulcer. Brit J Surg.

[7] Arnaud JP, Tuech JJ, Bergamaschi R, Pessaux P, Regenet N (2002). Laparoscopic suture closure of perforated duodenal peptic ulcer. Surg Laparosc Endosc Percutan Tech.

[8] Lau H (2004). Laparoscopic repair of perforated peptic ulcer: a meta-analysis. Surg Endosc.

[9] Cameron AM (2017). Current Surgical Therapy.

[10] Sanabria A, Villegas MI, Morales Uribe CH (2013). Laparoscopic repair for perforated peptic ulcer disease. Cochrane Database Syst Rev.

[11] Vakayil V, Bauman B, Joppru K (2019). Surgical repair of perforated peptic ulcers: laparoscopic versus open approach. Surg Endosc.

[12] Cirocchi R, Soreide K, Di Saverio S (2018). Meta-analysis of perioperative outcomes of acute laparoscopic versus open repair of perforated gastroduodenal ulcers. J Trauma Acute Care Surg.

[13] Quah GS, Eslick GD, Cox MR (2019). Laparoscopic repair for perforated peptic ulcer disease has better outcomes than open repair. J Gastrointest Surg.

[14] Lau WY, Leung KL, Kwong KH (1996). A randomized study comparing laparoscopic versus open repair of perforated peptic ulcer using suture or sutureless technique. Ann Surg.

[15] Cellan-Jones CJ (1929). A rapid method of treatment in perforated duodenal ulcer. Br Med J.

[16] Graham R (1937). The treatment of perforated duodenal ulcers. Surg Gynecol Obstetric.

[17] Alhaj Saleh A, Esquivel EC, Lung JT (2019). Laparoscopic omental patch for perforated peptic ulcer disease reduces length of stay and complications, compared to open surgery: a SWSC multicenter study. Am J Surg.

[18] Zhou C, Wang W, Wang J (2015). An updated meta-analysis of laparoscopic versus open repair for perforated peptic ulcer. Sci Rep.

[19] Mouly C, Chati R, Scotté M, Regimbeau JM (2013). Therapeutic management of perforated gastro-duodenal ulcer: literature review. J Visc Surg.

[20] Kim JH, Chin HM, Bae YJ, Jun KH (2015). Risk factors associated with conversion of laparoscopic simple closure in perforated duodenal ulcer. Int J Surg.

[21] Guadagni S, Cengeli I, Galatioto C (2014). Laparoscopic repair of perforated peptic ulcer: single-center results. Surg Endosc.

[22] Lunevicius R, Morkevicius M (2005). Risk factors influencing the early outcome results after laparoscopic repair of perforated duodenal ulcer and their predictive value. Langenbecks Arch Surg.

[23] Muller MK, Wrann S, Widmer J, Klasen J, Weber M, Hahnloser D (2016). Perforated peptic ulcer repair: factors predicting conversion in laparoscopy and postoperative septic complications. World J Surg.

[24] Song KY, Kim TH, Kim SN, Park CH (2008). Laparoscopic repair of perforated duodenal ulcers: the simple “one-stitch” suture with omental patch technique. Surg Endosc.

[25] Teoh AY, Chiu PW, Kok AS, Wong SK, Ng EK (2015). The selective use of laparoscopic repair is safe in high-risk patients suffering from perforated peptic ulcer. World J Surg.

[26] Zimmermann M, Hoffmann M, Laubert T, Jung C, Bruch HP, Schloericke E (2015). Conversion of laparoscopic surgery for perforated peptic ulcer: a single-center study. Surg Today.

[27] Lagoo S, McMahon RL, Kakihara M, Pappas TN, Eubanks S (2002). The sixth decision regarding perforated duodenal ulcer. JSLS.

[28] Kumar P, Khan HM, Hasanrabba S (2014). Treatment of perforated giant gastric ulcer in an emergency setting. World J Gastrointest Surg.

[29] Lee FY, Leung KL, Lai PB, Lau JW (2001). Selection of patients for laparoscopic repair of perforated peptic ulcer. Br J Surg.

